# Emerging Novel Therapies for the Treatment of Psoriasis: A Narrative Review

**DOI:** 10.7759/cureus.79693

**Published:** 2025-02-26

**Authors:** Alan D Kaye, Nicholas Thompson, Camille B Coreil, Lane S Amedio, Victoria A Rodriguez, Judy N Vu, Shahab Ahmadzadeh, Anusha Kallurkar, Taylor W Moss, Sahar Shekoohi, Giustino Varrassi

**Affiliations:** 1 Anesthesiology, Louisiana State University Health Sciences Center, Shreveport, USA; 2 Medicine, School of Medicine, Louisiana State University Health Sciences Center, Shreveport, USA; 3 Pain Medicine, Fondazione Paolo Procacci, Rome, ITA

**Keywords:** biological treatments, gut microbiome, il-21 inhibition, natural remedies, psoriasis, snora73, therapeutic advancements

## Abstract

Psoriasis is a chronic autoimmune and autoinflammatory disorder defined by abnormal skin cell turnover and inflammation, resulting in the formation of plaques on the skin. Although biologic therapies targeting interleukin (IL)-17 and IL-23 have significantly improved the treatment landscape for moderate-to-severe psoriasis, they are not effective for all patients. This highlights the need for additional therapeutic strategies. In recent years, exploring novel treatment avenues such as targeting IL-21, small nucleolar RNA (snoRNA) Snora73, the gut microbiome, and natural remedies have shown increasing promise in managing psoriasis. Interleukin-21 is a cytokine that plays a critical role in the differentiation and function of Th17 cells, which are central to the pathogenesis of psoriasis. Recent studies have demonstrated that neutralizing IL-21 with specific antibodies can help restore immune homeostasis, reducing disease severity and improving patient outcomes. Targeting IL-21 may be particularly beneficial for patients resistant to conventional therapies like IL-17 and IL-23 inhibitors. In addition to IL-21, snoRNA Snora73 has emerged as a novel target for psoriasis treatment. Snora73 regulates cell proliferation by interacting with miR-3074-5p and pre-B-cell leukemia homeobox 1 (PBX1), promoting abnormal cell turnover in psoriasis. The gut microbiome is increasingly recognized for its role in autoimmune diseases, including psoriasis. Imbalances in the microbiome have been linked to disease exacerbation, triggering systemic inflammation and altering immune responses. Moreover, various natural treatments have gained attention for their anti-inflammatory properties. These natural therapies could serve as adjuncts to existing treatments, offering a complementary approach that minimizes side effects while improving patient outcomes. Targeting IL-21, Snora73, and the gut microbiome, as well as utilizing natural treatments, may provide new opportunities for more effective, personalized management of psoriasis.

## Introduction and background

Psoriasis is a chronic inflammatory autoimmune disorder that primarily affects the skin of 3.0% of adults in the United States [1,2]. It is commonly characterized by papules and plaques with a silvery scaling caused by the unregulated proliferation of skin cells [1]. Psoriasis primarily affects the scalp, trunk, and extensor surfaces, such as the elbows or the knees [1,3]. While psoriasis occurs worldwide and affects both males and females, studies show that females tend to develop symptoms earlier [1,3]. It has been shown to have a bimodal distribution in age of onset with symptoms most commonly presenting in an individual’s 30s and 60s [3]. Individuals with a family history of psoriasis have increased incidence rates, further demonstrating the disease’s genetic component [4]. Due to the waxing and waning nature of the disease, psoriasis is often called the ‘undying disease’ [5]. This can potentially lead to depression and suicidal ideation [5]. Beyond its physical manifestations, psoriasis originates from complex pathogenesis that drives its chronic, relapsing nature.

The pathogenesis of psoriasis involves accumulations of Th17 immune cells that produce inflammatory mediators such as IL-17 and IL-23 cytokines [6]. These cytokines infiltrate the skin, leading to the proliferation of keratinocytes and epidermal hyperplasia, and are significantly increased in psoriatic patients [5,6]. The IL-23-IL-17A axis is pivotal in the pathogenesis of psoriasis, as evidenced by the success of biologic agents targeting IL-23 and IL-17A in psoriasis treatment [6]. Anti-IL17 and anti-IL23 monoclonal antibodies have been developed as treatment options for psoriasis [7]. However, these monoclonal antibodies have shown side effects. Upper respiratory infections, mild candida albicans mucocutaneous infections, and new inflammatory bowel disease (IBD) outbreaks have all been associated with anti-IL17 [7]. Anti-IL23 has been associated with an increased chance of cerebrovascular accident, though this data has been placed under scrutiny [7]. These repercussions prompt further exploration of alternative treatment options.

Recent advances in psoriasis treatment have introduced a range of novel therapeutic options targeting diverse biological mechanisms. Interleukin-21 has been shown to contribute to psoriasis pathogenesis via the proliferation of CD4+ T cells, increasing function and differentiation of Th17 cells, and downregulation of regulatory T cell (Treg) cell differentiation [6]. Thus, IL-21 inhibitors show potential as a therapeutic option due to their ability to interfere with the immunological pathway of psoriasis [5]. Another potential treatment route is through small nucleolar RNA (snoRNA). These are noncoding RNA that could potentially modify ribosomal RNA [8]. A specific snoRNA, Snora73, was highly expressed in psoriasis and further promotes its progression [8]. Zhang et al. provided evidence that the knockdown of Snora73 leads to the suppression of cell proliferation and migration, contributing to its role as a treatment option in psoriasis [8]. Emerging research also highlights the potential of the gut microbiome, where restoring one’s microbial balance can induce improvements to systemic inflammation and immune responses linked to psoriasis [9]. Natural treatments, such as compounds like luteolin, piperine, and glycyrrhizin, complement these innovations and provide anti-inflammatory benefits with fewer side effects reported [10]. These novel therapies signal a paradigm shift toward more personalized and integrative care for psoriasis. In this review, we will focus on these emerging therapies and their implications for advancing the treatment of psoriasis.

## Review

IL-21, an alternative inhibition target

Interleukin-21 has recently gained attention as a potential therapeutic target in psoriasis due to its role in promoting inflammatory immune responses, mainly through modulation of T helper cell subtypes, such as Th17 and Tregs [6]. Current therapeutic approaches for psoriasis often target cytokines like IL-17 and IL-23 to alleviate inflammation. However, IL-21 presents an alternative route with specific promise due to its distinct effects on immune cell differentiation and cytokine expression​ [11]. Studies have identified IL-21 as a key factor in psoriasis pathology, with elevated levels associated with the severity of psoriasis, promoting an inflammatory environment by encouraging Th17 differentiation while inhibiting Treg cells, which typically act to moderate immune responses​ [6,12]. This Th17/Treg imbalance leads to exacerbated inflammatory responses, further driving the progression of psoriasis​ [13]. For instance, Shi et al. investigated IL-21 expression in moderate-to-severe plaque psoriasis patients. They found increased IL-21 and IL-21 receptor (IL-21R) expression in lesional skin and peripheral blood samples of psoriasis patients​ [6]. The study illustrated that IL-21 induced Th17 differentiation, enhanced expression of pro-inflammatory cytokines IL-17A and IL-22, and suppressed Treg cell differentiation by downregulating transcription factor Foxp3 expression.​ [6].

In psoriasis, Th17 cells produce IL-17A and IL-22, cytokines that lead to keratinocyte proliferation and contribute to the thick, scaly plaques characteristic of the disease​ [11]. The imbalance between Th17 and Treg cells is significant; Treg cells typically counteract the inflammatory activity of Th17 cells, but IL-21 disrupts this balance by hindering Treg functionality​ [11]. Wang et al. found that elevated serum levels of IL-21 and an increase in IL-21-producing CD4+ T cells, particularly Th17 cells, correlated with higher psoriasis area and severity index (PASI) scores, which measure psoriasis severity. Their study emphasized IL-21’s role in enhancing Th17 proliferation and differentiation, demonstrating that the presence of IL-21 increases the population of Th17 cells relative to Treg cells, creating a feedback loop that sustains chronic inflammation​ [11]. This highlights IL-21’s potential as a therapeutic target, as modulating IL-21 levels may help restore immune balance by curbing Th17-driven inflammation and allowing Treg cells to regain their regulatory role​ [13].

Beyond psoriasis, IL-21’s contribution to autoimmune diseases further highlights its potential as a therapeutic target​ [13]. Ren et al. reviewed the evidence of IL-21’s role in autoimmune pathology, emphasizing its involvement in T-cell and B-cell interactions that sustain inflammation. For example, IL-21's interaction with B cells enhances the production of autoantibodies, which are key markers of autoimmune diseases. In rheumatoid arthritis, IL-21 has been shown to act on synovial macrophages, fibroblasts, and T cells within the joint, promoting local inflammation and tissue degradation​ [13]. These findings suggest that targeting IL-21 may offer a broader application in autoimmune disease treatment by interrupting the pathogenic cycles driven by IL-21-induced immune responses​ [13].

The advent of IL-21 inhibitors provides a promising therapeutic approach. However, IL-21’s multifaceted role in immune regulation poses challenges for therapeutic targeting​ [13]. Interleukin-21 is involved in autoimmune inflammation and contributes to antiviral and antitumor immune responses​ [13]. Thus, complete inhibition of IL-21 could carry risks of weakening the immune system’s ability to combat infections or tumors​ [13].

The therapeutic challenge is to achieve a balance in which IL-21 activity is sufficiently reduced to alleviate autoimmune pathology without significantly impairing immune defenses​ [13]. One approach to achieving this balance might involve selective inhibition of IL-21 signaling pathways specific to Th17 cells while sparing pathways that support antiviral immunity​ [13].

In conclusion, IL-21 is a promising target for novel psoriasis therapies, given its central role in modulating the balance between pro-inflammatory and regulatory T-cell populations​ [13,14]. The cytokine’s involvement in T-cell differentiation and immune regulation in various autoimmune diseases underscores its therapeutic relevance​ [13]. Developing IL-21 inhibitors or combination therapies may offer new hope for psoriasis patients, particularly those nonresponsive to current therapies targeting IL-17 and IL-23​ [11]. By focusing on IL-21, research can aim to disrupt the chronic inflammatory loop characteristic of psoriasis, restore immune equilibrium, and reduce the disease’s burden on patients’ lives​ [6]. Future studies can prioritize understanding IL-21’s specific signaling mechanisms in psoriasis, enabling precise therapeutic interventions that maximize efficacy and minimize adverse effects.

Snora73 and psoriasis 

A promising new approach for psoriasis treatment involves more precise, targeted disruption of snoRNA. This regulatory molecule plays a critical role in the disease progression of psoriasis. The snoRNAs are a class of non-coding RNAs that are most notably involved in the modification and maturation of ribosomal RNA [15]. They guide chemical modifications of rRNA, such as methylation and pseudoridylation, both of which are essential in the proper formation and function of ribosomes [15]. However, more recent research has revealed broader functional implications of small nuclear RNAs (snRNAs) in gene regulation involving alternative splicing, mRNA stability, and chromatin remodeling, impacting cellular processes far beyond ribosomal biogenesis [15]. The snoRNAs' ability to modify gene regulation reflects their functional diversity and impact on cellular homeostasis and function. Disruption of snoRNA expression and function has been linked to various neurodevelopmental, cancerous, and autoimmune diseases, further highlighting their importance in maintaining cellular integrity and function [15]. Particularly in the context of psoriasis, modifications within snoRNA activity have the potential to amplify keratinocyte hyperproliferation and inflammatory responses, both of which are notable features of the disease [8].

Snora73k, The Molecular Sponge

One snoRNA, Snora73, acts as a “molecular sponge” for a particular microRNA, miR-3074-5p [8]. MicroRNA are noncoding RNAs that are involved in the regulation of mRNA expression [8]. The pre-B-cell leukemia homeobox 1 (PBX1), is a transcription factor that plays an important role in cell cycle regulation and characteristically promotes cellular proliferation [8]. Under normal conditions, miR-3074-5p can bind to PBX-1, suppressing its expression and leading to a more regulated cell cycle progression [8]. However, when Snora73 interacts with miR-3074-5p, it inhibits the binding of miR-3074-5p to its target PBX-1 mRNA. In blocking this interaction, suppression of PBX-1 is relieved, allowing for an enhanced rate of cellular proliferation [8]. Elevated PBX-1 expression can promote keratinocyte activity, leading to inflammation and thickening of the skin, resulting in the plaques and scales characteristically observed in psoriasis patients [8]. The Snora 73-mediated regulation of PBX-1 expression introduces a pathway that may explain how non-coding RNA activity contributes to the dysregulation of inflammation and keratinocyte proliferation in the progression of psoriasis [8]. Modulation of this pathway provides a possible regulation mechanism for psoriasis's inflammatory and hyperproliferative characteristics. Identifying Snora73 as a crucial regulator in the expression factors involved in psoriasis pathology offers a promising new avenue of therapeutic intervention. By targeting the binding of Snora73 with miR-3074-5p, excessive production of PBX-1 may be reduced, curbing the erratic keratinocyte proliferation notable in psoriasis [8].

While traditional psoriasis treatments typically focus on more broad modulation of inflammatory pathways and immune response, this approach targets specific molecular interactions at the post-transcriptional level, offering more targeted and refined treatment [16]. Furthermore, by directly addressing post-transcriptional dysregulation of PBX1 expression, patients who experience chronic or treatment-resistant psoriasis could potentially experience a reduction in both the severity and frequency of psoriatic episodes. While research in this area is relatively novel, future studies could explore broader applications, including combinational therapy integration with existing psoriasis treatment, to examine possible synergetic effects of treatment that may provide better psoriatic patient outcomes. 

Exploring the gut microbiome role in psoriasis

Insights From Western and Traditional Chinese Medicine

Psoriasis, one of the most common mixed autoimmune and inflammatory skin diseases, appears to correlate with a diverse gut microbiome. The human gut microbiota is a complex and diverse community mainly comprising bacteria from the phyla Firmicutes, Bacteroidetes, Actinobacteria, and Proteobacteria, which support nutrient absorption, immune regulation, and pathogen defense. At the same time, disruptions can compromise gut homeostasis and immune function, potentially leading to chronic inflammatory diseases [17]. Psoriasis patients show that the abundance of Firmicutes and Actinobacteria increases, while the abundance of Bacteroidetes decreases [17,18]. These trends suggest that the diversity of the gut microbial community becomes homogenous as PSO progresses [18]. Western medicine and traditional Chinese approaches have tried to improve psoriasis symptoms by targeting gut microbiota composition and function [19]. Western medicine encompasses medications that interact with gut microbiota directly or indirectly, including IL-23 inhibitors like guselkumab and IL-17 inhibitors such as secukinumab and ixekizumab. According to clinical evidence, the abundance of *Roseburia*, *Lachnoclostridium*, *Bacteroides vulgatus*, *Anaerostipe*s, and *Escherichia/Shigella* increases with IL-23 inhibitor administration, while IL-17 inhibitor administration significantly increases the levels of* Bacteroides stercoris* and *Parabacteriodes merdae*, and decreases the levels of *Blautia* and *Roseburia *[17]. Alterations in microbial diversity may influence intestinal inflammatory responses. Furthermore, studies indicate that psoriasis patients with ustekinumab, compared to untreated individuals, showed increased proportions of some bacterial groups that are known to produce anti-inflammatory metabolites like butyrate and medium-chain fatty acids (MCFAs), which can reduce intestinal oxidative stress and modulate the Th17/Treg balance [17].

Traditional Chinese Medicine and the Gut Microbiome

In China, traditional Chinese medicine has been applied in psoriasis treatment and has demonstrated significant efficacy in alleviating symptoms. Recently, animal studies have begun to elucidate the effect of individual herbal ingredients and compound formulas on inflammatory psoriasis models. For instance, rosin isolated from frankincense, when administered to imiquimod (IMQ)-induced mice, was shown to reduce inflammation by inhibiting IL-23/IL-17 axis-related cytokines and, through 16S rRNA sequencing, demonstrated an ability to reshape the gut microbiota [17]. Additionally, curcumin has been shown to decrease levels of pro-inflammatory factors like IFN-γ and IL-17 in psoriasis patients, producing anti-inflammatory effects and inducing microbiome changes associated with inflammation regulation [20]. Specifically, studies have found that *Ligilactobacillus* and *Anaeroplasma *correlate positively with psoriasis-associated cytokines IL-6, IL-17A, IL-22, and IL-23, while *Rikenella*, *Alistipes*, and *Mucispirillum* are negatively correlated [17]. Another compound, quercetin, is a widely recognized polyphenolic flavonoid found in Chinese herbs with anti-inflammatory, antioxidant stress, and antibacterial properties. Recent studies have highlighted quercetin’s impact on gut microbiota, increasing beneficial bacteria such as *Bifidobacterium* and reducing harmful species like *Clostridium difficile* [17]. This shift contributes to gut health and immune regulation. Quercetin also shows promise in alleviating psoriasis symptoms by lowering pro-inflammatory cytokines and oxidative stress markers, thereby improving clinical signs such as erythema, scaling, and skin thickness [17].

Further research is needed to explore the mechanisms underlying quercetin’s modulation of the gut-skin axis, especially in psoriasis. Regulating gut microbiota through medicinal or natural treatments has emerged as a significant research focus to alleviate disease symptoms, but limited experimental studies and a lack of data from diverse populations remain a challenge. In addition to targeting gut microbiota, various natural treatments have shown promise in managing psoriasis symptoms.

Natural compounds as therapeutic agents for psoriasis

Several natural product-derived compounds, such as luteolin, piperine, and glycyrrhizin, have been presented as new potential therapies for alleviating symptoms through anti-inflammatory and immunomodulatory mechanisms. Luteolin is a naturally occurring vegetable, fruit, and medicinal herb flavonoid [21]. It is synthesized in plants through the phenylpropanoid pathway and is known for its wide-ranging biological effects, including antioxidant, anti-inflammatory, anticancer, hepatoprotective, and neuroprotective effects [10]. In psoriasis treatment, luteolin has shown strong anti-inflammatory properties in keratinocytes by inhibiting TNF-α-induced production of inflammatory mediators, including IL-6, IL-8, and VEGF. It also reduces TNF α-induced phosphorylation, nuclear translocation, and DNA binding, which are all crucial steps in inflammation-related transcription. Furthermore, luteolin suppresses NF-κB subunit mRNA expression and effectively curtails keratinocyte proliferation, a characteristic feature of psoriatic lesions [10].

Piperine, the principal alkaloid derived from *Piper longum*, has also demonstrated considerable pharmacological potential by inhibiting the phosphorylation of the signal transducer and activator of transcription (STAT3) [22]. By inhibiting STAT3 phosphorylation, piperine effectively ameliorates psoriatic skin lesions and reduces inflammatory cell infiltration by decreasing the expression of cytokine and chemokines associated with psoriasis [10].

Glycyrrhizin, also known as glycyrrhizin acid, is a compound found in licorice that imparts its characteristic sweetness. The anti-inflammatory, anti-carcinogenic, and antiviral effects of glycyrrhizin are attributed to its active metabolite, glycyrrhetenic acid. Its therapeutic potential for psoriasis is linked to its ability to target Toll-like receptor (TLR-4) and inhibit the protease TMPRSS2, which plays a role in viral entry. Glycyrrhetenic acid has been shown to inhibit the expression of IL-17A and IFN-γ, and its capacity to suppress cell proliferation and reverse inflammatory markers suggests it may alleviate psoriasis by modulating the immune response and keratinocyte proliferation through the IL-17A/SIRT1/STAT3 signaling axis [10].

Other natural compounds, such as kaempferol found in plants, punicalagin found in pomegranates, shikonin found in roots of plants, and genistein found in soybeans, have shown potential in addressing psoriasis by targeting various immune response pathways, including JAK-STAT, CEBPD, and SPK2; however, their efficacy in providing definitive therapeutic benefits for psoriasis remains unproven [23-26]. As previously mentioned, curcumin, derived from turmeric, exerts its effects by regulating and repressing Th-17-related inflammatory factors, as demonstrated in studies involving mice. Additionally, the observed increase in *Lactobacillus* may be attributed to a reduction in gut microbiota diversity. Notably, the abundance of* Rikenella* was also promoted in the curcumin group of mice; *Rikenella* has been shown to facilitate the catabolism of polysaccharides, leading to the production of short-chain fatty acids (SCFAs), which play a crucial role in maintaining intestinal barrier function and overall gut health [27]. Given the association between psoriasis and the gut microbiome, it is evident that diet influences the composition and stability of gut microbiota [28].

The Mediterranean diet, characterized by a high intake of fruits, vegetables, polyphenol-rich foods, fiber, and vitamins, exemplifies a model of healthy eating [28,29]. The antioxidant and anti-inflammatory properties inherent in this diet can alleviate psoriasis symptoms and have been found to have a negative correlation with the severity of the condition [30]. A cross-sectional observational study of psoriasis patients indicated that adherence to the Mediterranean diet can increase gut microbiota diversity [17]. Therefore, dietary therapy warrants consideration as a potential treatment strategy (Table 1).

**Table 1:** Potential natural derived therapies for psoriasis TMPRSS2: Transmembrane protease, serine 2; IL: Interleukin; IFN-y: Interferon-y; NF-κB: Nuclear factor-kappa B; TNF-α: Tumor necrosis factor-alpha; JAK-STAT: Janus kinase-signal transducer and activator of transcription; STAT-3: Signal transducer and activator of transcription 3, PASI: Psoriasis area and severity index

Therapy	Target	Result	Reference No.
Rosin	Inhibit IL-23/IL-17 axis-related cytokines	Reduction in inflammation	[17]
	Modify gut microbiota		
Curcumin	Reduce IFN-γ, IL-17	Reduction in inflammation	[20]
	Modify gut microbiota		
Quercetin	Reduce TNF-α, IL-6, IL-17	Reduced PASI score	[17]
	Inhibit NF-κB signaling	Decreased hyperkeratosis	
	Modify gut microbiota	Improved psoriasis symptoms	
Luteolin	Inhibit TNF-α-induced inflammatory mediators	Reduces keratinocyte proliferation	[10,21]
	Suppress NF-κB subunit mRNA expression		
Piperine	Inhibit phosphorylation of STAT3	Improvement in psoriatic skin lesions	[10,22]
Glycyrrhetinic acid	Inhibit TMPRSS2	Suppressed keratinocyte proliferation	[10]
	Inhibit expression of IL-17A, IFN-γ	Reversed IL-17A inflammatory markers	
Kaempferol	Decrease NF-κB signaling	Reduced psoriatic skin lesions	[23]
		Reduction in inflammation	
Punicalagin	Decrease IL-6 and TNF-α secretion	Reduced JAK-STAT activation	[25]
		Reduction in inflammation	
Shikonin	Inhibit NF-κB pathway	Reduction in inflammation	[24]
	Inhibit phosphorylation of STAT3		
Mediterranean diet	Reduced consumption of red meat and increased polyunsaturated fats	Improved psoriasis symptoms	[30]
	Modify gut microbiota		

Discussion

Psoriasis remains a challenging condition to manage, but emerging therapies offer new hope for more effective and personalized treatment options. Current treatments targeting the biologic agents of IL-17 and IL-23 and their pathways have paved the way in management for many patients but still demonstrate limitations in their effectiveness as a treatment option. Thus, new novel treatments are a topic in current research regarding psoriasis. This review highlights the potential of several novel therapeutic avenues that could significantly advance the treatment spectrum for psoriasis. Among these, IL-21 inhibitors are of interest due to their ability to module immune balance by reducing Th17-driven inflammation and enhancing regulatory T-cell activity. These mechanisms are essential in the pathogenesis of psoriasis and thus may serve as an effective treatment option for cases that do not respond to IL-17 or IL-23 inhibitors. A novel monoclonal antibody is also suggested in the therapy [31].

Furthermore, snoRNA Snora73 has also been shown to play a role in the pathogenesis of psoriasis. Evidence has shown increased levels of Snora73 in psoriasis patients, where psoriasis cells demonstrated increased viability and migration. Research on this pathway has shown promising results, with evidence that knockdown of Snora73 reduces keratinocyte hyperplasia and disease progression. Similarly, the gut microbiome has been revealed to be an area of focus in its potential as a novel therapy in psoriasis. Research has shown how dysbiosis plays a role in systemic inflammation and immune dysregulation in psoriasis. Restoring microbial balance with probiotics, different dietary practices, and other interventions may offer another approach to the management of psoriasis.

Natural compounds have also been of interest in the research of new therapeutic options in the treatment of psoriasis. Such agents include luteolin, piperine, quercetin, and curcumin. Through evidence, these compounds exhibited anti-inflammatory, antioxidant, and immunomodulatory properties. They have also shown fewer side effects compared to other treatments. Their ability to target multiple pathways involved in psoriasis pathogenesis has made them an attractive treatment option for patients with this disease.

These advancements collectively represent a paradigm shift toward personalized and integrative psoriasis management. The benefits of a personalized approach go beyond minimizing the side effects of treatment. Special attention can be paid, especially in the elderly, to prevent diagnostic confusion with other conditions like bacterial infections [32]. By integrating evidence from immunology, genetics, microbiology, and natural medicine, these therapies have the potential to address the diverse needs of psoriasis patients and improve their quality of life. Nevertheless, several challenges remain, including clinical trials to evaluate the effectiveness and safety of these emerging treatments. Further research is needed to gain a deeper understanding of the therapies themselves, how they interact with current treatments, and how they could be optimized for specific patient subgroups.

## Conclusions

In summary, there are a plethora of potential avenues available for the treatment of psoriasis. Going forward, more research will be needed to move towards human trials, though well-studied remedies like following the Mediterranean diet could be applied now. Integrating novel therapies into clinical practice could redefine the standard of care for psoriasis. With continued innovation and collaboration across disciplines, psoriasis management could become more effective, comprehensive, and tailored to individual patient needs. By addressing the limitations of current therapies and embracing these new strategies, clinicians and researchers can provide renewed hope for patients living with the burdensome condition of psoriasis.

## References

[1] Nair PA, Badri T (2024). Psoriasis. StatPearls Publishing.

[2] Armstrong AW, Mehta MD, Schupp CW, Gondo GC, Bell SJ, Griffiths CE (2021). Psoriasis prevalence in adults in the United States. JAMA Dermatol.

[3] Raharja A, Mahil SK, Barker JN (2021). Psoriasis: a brief overview. Clin Med (Lond).

[4] Rendon A, Schäkel K (2019). Psoriasis pathogenesis and treatment. Int J Mol Sci.

[5] Wang J, Wang C, Liu L (2023). Adverse events associated with anti-IL-17 agents for psoriasis and psoriatic arthritis: a systematic scoping review. Front Immunol.

[6] Shi Y, Chen Z, Zhao Z (2019). IL-21 induces an imbalance of Th17/Treg cells in moderate-to-severe plaque psoriasis patients. Front Immunol.

[7] Potestio L, Martora F, Lauletta G, Vallone Y, Battista T, Megna M (2024). The role of interleukin 23/17 axis in psoriasis management: a comprehensive review of clinical trials. Clin Cosmet Investig Dermatol.

[8] Zhang L, Guo H, Zhang X (2024). Small nucleolar RNA Snora73 promotes psoriasis progression by sponging miR-3074-5p and regulating PBX1 expression. Funct Integr Genomics.

[9] Buhaș MC, Gavrilaș LI, Candrea R, Cătinean A, Mocan A, Miere D, Tătaru A (2022). Gut microbiota in psoriasis. Nutrients.

[10] Lee YG, Jung Y, Choi HK, Lee JI, Lim TG, Lee J (2024). Natural product-derived compounds targeting keratinocytes and molecular pathways in psoriasis therapeutics. Int J Mol Sci.

[11] Wang Y, Wang LL, Yang HY, Wang FF, Zhang XX, Bai YP (2016). Interleukin-21 is associated with the severity of psoriasis vulgaris through promoting CD4+ T cells to differentiate into Th17 cells. Am J Transl Res.

[12] Varkey R, Du Q, Karnell JL (2019). Discovery and characterization of potent IL-21 neutralizing antibodies via a novel alternating antigen immunization and humanization strategy. PLoS One.

[13] Ren HM, Lukacher AE, Rahman ZS, Olsen NJ (2021). New developments implicating IL-21 in autoimmune disease. J Autoimmun.

[14] Yang L, Anderson DE, Baecher-Allan C (2008). IL-21 and TGF-beta are required for differentiation of human T(H)17 cells. Nature.

[15] Bratkovič T, Božič J, Rogelj B (2020). Functional diversity of small nucleolar RNAs. Nucleic Acids Res.

[16] Greb JE, Goldminz AM, Elder JT (2016). Psoriasis. Nat Rev Dis Primers.

[17] Zou X, Zou X, Gao L, Zhao H (2024). Gut microbiota and psoriasis: pathogenesis, targeted therapy, and future directions. Front Cell Infect Microbiol.

[18] Zhao H, Shang L, Zhang Y (2024). IL-17A inhibitors alleviate psoriasis with concomitant restoration of intestinal/skin microbiota homeostasis and altered microbiota function. Front Immunol.

[19] Zhang S, Wang J, Liu L (2022). Efficacy and safety of curcumin in psoriasis: preclinical and clinical evidence and possible mechanisms. Front Pharmacol.

[20] Ghoushi E, Poudineh M, Parsamanesh N, Jamialahmadi T, Sahebkar A (2024). Curcumin as a regulator of Th17 cells: unveiling the mechanisms. Food Chem (Oxf).

[21] Zhu M, Sun Y, Su Y (2024). Luteolin: a promising multifunctional natural flavonoid for human diseases. Phytother Res.

[22] Lu H, Gong H, Du J (2023). Piperine ameliorates psoriatic skin inflammation by inhibiting the phosphorylation of STAT3. Int Immunopharmacol.

[23] Alrumaihi F, Almatroodi SA, Alharbi HO, Alwanian WM, Alharbi FA, Almatroudi A, Rahmani AH (2024). Pharmacological potential of kaempferol, a flavonoid in the management of pathogenesis via modulation of inflammation and other biological activities. Molecules.

[24] Guo C, He J, Song X (2019). Pharmacological properties and derivatives of shikonin — a review in recent years. Pharmacol Res.

[25] Xu J, Cao K, Liu X, Zhao L, Feng Z, Liu J (2021). Punicalagin regulates signaling pathways in inflammation-associated chronic diseases. Antioxidants (Basel).

[26] Goh YX, Jalil J, Lam KW, Husain K, Premakumar CM (2022). Genistein: a review on its anti-inflammatory properties. Front Pharmacol.

[27] Cai Z, Wang W, Zhang Y, Zeng Y (2023). Curcumin alleviates imiquimod-induced psoriasis-like inflammation and regulates gut microbiota of mice. Immun Inflamm Dis.

[28] Rinninella E, Cintoni M, Raoul P (2019). Food components and dietary habits: keys for a healthy gut microbiota composition. Nutrients.

[29] Cannataro R, Fazio A, La Torre C, Caroleo MC, Cione E (2021). Polyphenols in the Mediterranean diet: from dietary sources to microRNA modulation. Antioxidants (Basel).

[30] Aryanian Z, Asghari M, Zanousi PP, Ghadimi R, Kebria AS, Hatami P (2024). Adherence to the Mediterranean diet in patients with psoriasis and its relationship with the severity of the disease: a case-control study. Health Sci Rep.

[31] Kerut CK, Wagner MJ, Daniel CP (2023). Guselkumab, a novel monoclonal antibody inhibitor of the p19 subunit of IL-23, for psoriatic arthritis and plaque psoriasis: a review of its mechanism, use, and clinical effectiveness. Cureus.

[32] Arshad M, Kumar A, Ur Rehman MS, Tito Rodriguez PR, Varrassi G (2024). Atypical mycobacterial infection mimicking psoriasis in an elderly patient: diagnostic challenges and management. Cureus.

